# Transforming Computed Energy Landscapes into Experimental Realities: The Role of Structural Rugosity

**DOI:** 10.1002/anie.202006939

**Published:** 2020-09-02

**Authors:** Riccardo Montis, Roger J. Davey, Sarah E. Wright, Grahame R. Woollam, Aurora J. Cruz‐Cabeza

**Affiliations:** ^1^ Department of Chemical Engineering and Analytical Science The University of Manchester Oxford Road Manchester M13 9PL UK; ^2^ Novartis Pharma AG 4002 Basel Switzerland

**Keywords:** computational chemistry, crystal structure prediction, crystallization, polymorphism, surface rugosity

## Abstract

We exploit the possible link between structural surface roughness and difficulty of crystallisation. Polymorphs with smooth surfaces may nucleate and crystallise more readily than polymorphs with rough surfaces. The concept is applied to crystal structure prediction landscapes and reveals a promising complementary way of ranking putative crystal structures.

## Graphical abstract

The link between structural surface rugosity and ease of crystallisation has been explored (see picture). Smooth polymorphs were found to crystallise more readily than rough polymorphs. A code was written to compute crystal rugosity and applied to crystal structure prediction calculations, thus affording a new way of linking landscapes to experimentation.

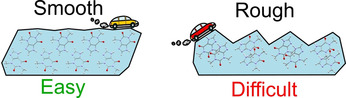

Perhaps one of the most enduring problems associated with the solid‐state chemistry of pharmaceutical materials remains the issue of polymorphism, where an active ingredient may adopt a number of different crystal structures.[^1^, ^2^] Typically the challenge here is to discover all polymorphs of a new active molecule suitable for production and formulation. Historically these issues have been addressed experimentally by performing rigorous screening tests, often involving hundreds of experiments, in order to discover all the possible forms. Such experimentation is increasingly being supported by state of the art crystal structure prediction (CSP) as a means of checking potential structures against observed ones to ensure that none are missed.[^3^, ^4^, ^5^]

CSP has evolved tremendously in the last twenty years or so to the point where this technique is now routinely used in industry.[^3^, ^4^, ^5^] A major problem of CSP energy landscapes, however, is that they over‐generate plausible crystal structures that are never realised experimentally.^[6]^ Additionally it is clear that beyond relative energy criteria we have no way of assessing which in silico structures might be accessible experimentally. Overcoming this limitation and hence realising the full potential of CSP, requires a link to be established between the kinetic processes of nucleation and crystal growth, which often dominate experiments, and the purely structural (molecular packing) outputs that come from CSP. In this communication we expand on recently published work to demonstrate that the surface roughness of potential crystal faces may hold the key to establishing such a link.[^7^, ^8^]

As a starting point we note that one central parameter common to both nucleation and growth processes is the interfacial tension. In nucleation theory this appears as a thermodynamic factor, which dictates the concentration of critical nuclei existing under certain conditions of supersaturation and temperature. In crystal growth it appears as the edge energy controlling the distance between spiral steps in dislocation‐controlled growth and the concentration of 2D nuclei in growth controlled by surface nucleation. The results of two recent studies are of relevance here. In the first it was reported that through neat grinding the stability of polymorphic forms could be reversed^[7]^ while in the second the existence of a growth dead zone,^[8]^ seen in many molecular materials, was confirmed. Of importance is that both of these features could be interrelated to molecular‐scale surface rugosities calculated from crystal morphological data.

The former of these observations is not unexpected since, given the Ostwald–Freundlich relationship between crystal size and solubility it follows that for two polymorphs there may indeed be a transition size at which the relative stability of forms switches.^[9]^ Belenguer et al.^[7]^ have shown that this transition goes from polymorphs with rough surfaces to those with smooth surfaces, suggesting that forms with rough surfaces have higher interfacial tensions than forms with smooth ones. In transferring this idea to nucleation we infer that the concentration C* of nuclei would be greater for forms with lower values of interfacial tension so that nucleation is favoured for those forms which on balance have smooth surfaces. As far as growth of these nuclei is concerned, the dead zone review of Liu et al. shows that growth too is linked to roughness with rough surfaces potentially exhibiting zero growth at low supersaturation.^[8]^ Thus we might argue that growth is also favoured by smooth surfaces, at least at low supersaturations.^[10]^ A combination of these arguments leads us quite naturally to enquire whether or not this simple concept of surface roughness actually holds the key to linking structural features to kinetic pathways, by suggesting that increasing rugosity of forms leads to reduced rates of both their nucleation and growth. In order to explore this idea, we have performed crystal surface rugosity calculations on a number of datasets.

To quantify the surface rugosity of each (*hkl*) face (*R* ^(*hkl*)^_depth_) of a crystal, we calculate the degree of interpenetration between two consecutive crystal (*hkl*) layers along a particular crystal direction using the method of Bryant, Maloney and Sykes.^[11]^ Briefly, *R* ^(*hkl*)^_depth_ is calculated as the distance between the highest atom in a given (*hkl*) crystal slice and the average plane between that slice and its consecutive slice. We note that the sign of the rugosity parameter simply refers to whether the surfaces are interpenetrated (negative) or separated (positive) by the value *R* ^(*hkl*)^_depth_. Thus when referring to rugosity in this article, the degree of interpretation is of interest here and it is linked to the roughness of the surface and the values of *R* ^(*hkl*)^_depth_ are discussed in absolute terms (for example a surface with a *R* ^(*hkl*)^_depth_ value of −2.8 Å has a larger rugosity than a surface with a *R* ^(*hkl*)^_depth_ value of −0.5 Å, and a surface with a *R* ^(*hkl*)^_depth_>0 Å is considered perfectly smooth). We have written a python program using the Cambridge Structural Database (CSD)^[12]^ python API which, for the crystal of interest, computes the BFDH morphology and provides the (*hkl*) values for its dominant faces,[^13^, ^14^, ^15^] the *d*‐spacings (*d* ^(*hkl*)^, in Å), the morphological importance for those faces (*w* ^(*hkl*)^) and their minimum rugosity value ($R_{min-depth}^{\left(hkl\right)}$ in Å). The minimum rugosity value is assessed from four independent calculations of rugosity for slices created at the same (*hkl*) value with their origins shifted by various fractions of *d* ^(*hkl*)^; for each (*hkl*) plane, the lowest value of all computed *R* ^(*hkl*)^_depth_ is then taken ($R_{min-depth}^{\left(hkl\right)}$ , see ESI). An overall normalised crystal rugosity value is then defined for a given crystal as the sum of the minimum rugosities of each (*hkl*) face normalised by the face *d*‐spacing and weighted by its BFDH morphological importance. The normalised crystal rugosity ${\bar{R}}_{depth}^{N}$ is then calculated as:

(1) $${\bar{R}}_{depth}^{N}=\sum_{\left(hkl\right)}w^{\left(hkl\right)}R_{min-depth}^{\left(hkl\right)}/d^{\left(hkl\right)}$$

The summation is carried out over all BFDH faces with negative *R*_min‐depth_^(*hkl*)^, since those are the faces displaying corrugation. We note that normalising by the *d*‐spacing allows for comparison across compounds of different molecular sizes.

Next, we set out to contextualise such rugosity values for molecules known to be polymorphic and appearing in the CSD. This was achieved by calculating the normalised crystal rugosities for 5611 crystal structures belonging to 2559 polymorphic families. For each family, the difference between the normalised crystal rugosities of the smoothest and the roughest polymorphs was then calculated (${|\Delta \bar{R}}_{depth}^{N}|$ ). The distribution of such difference is given in Figure 1 which shows that in 74 % of polymorphic families values of ${\Delta \bar{R}}_{depth}^{N}$ differ by a maximum of 0.08, 96 % by 0.17 and over 98 % by a maximum of 0.20. This histogram reveals the maximum difference in normalised crystal rugosities within polymorphic families in the CSD and these statistics have the potential to be used for interpreting CSP landscapes by limiting the “realisable forms” to those with lowest rugosities (and lying within 0.20 normalised crystal rugosity values from the smoothest polymorph). To test the utility of this conclusion and its application to polymorphic materials we have constructed a dataset of polymorphic pairs which have been reported to be either easy or difficult to crystallise (Table 1).

**Figure 1:**
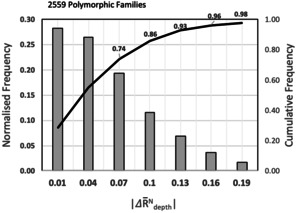
Distribution of the maximum difference in normalised crystal rugosity for 2559 polymorphic families (5611 crystal structures) in the CSD.

**Table 1:** Average normalised crystal rugosities for polymorphic systems with a form hard to produce and a more common form that is easier to crystallise.

	Easy		Hard		${\Delta \bar{R}}_{depth}^{N}\left[eh\right]$ ^[a]^
	form	${\bar{R}}_{depth}^{N}$	form	$\;{\bar{R}}_{depth}^{N}$	
rotigotine^[3][b]^	I	−0.402	II^[c]^	−0.372	−0.030
dapsone^[16]^	III	−0.109	V^[c]^	−0.106	−0.003
*p*ABA^[17]^	α	−0.061	β^[d]^	−0.091	0.030
aspirin[^18^, ^19^]	I^[c]^	−0.070	II	−0.121	0.051
curcumin^[20][b]^	I^[c]^	−0.233	II	−0.288	0.055
axitinib^[21][b]^	XXV	−0.115	XLI^[c,e]^	−0.170	0.055
5‐Br‐aspirin[^22^, ^23^]	I^[c]^	−0.061	II	−0.117	0.056
paracetamol^[24]^	I^[c]^	−0.153	II	−0.216	0.063
carbamazepine^[25]^	III^[c]^	−0.125	V	−0.193	0.068
theophylline^[26]^	II	−0.025	IV^[c]^	−0.161	0.136
ritonavir^[27][b]^	I	−0.114	II^[c]^	−0.265	0.151

[a] ${\Delta \bar{R}}_{depth}^{N}\left[eh\right]=\;{\bar{R}}_{depth}^{N}\left(easy\right)-{\bar{R}}_{depth}^{N}\left(hard\right)$ . [b] Conformational polymorphs. [c] Thermodynamically stable form under ambient conditions. [d] Thermodynamically stable at low temperature. [e] Formed by conversion from a solvate.

The attribution of a given polymorph to the category “difficult” is made on the basis of it not being obtainable from straightforward solution crystallisation. This definition includes polymorphic forms that can only be obtained by solid‐solid transformations, solvent‐mediated transformations or by desolvation as well as conditions which require the presence of impurities or high pressure (see ESI). By contrast, a polymorph is noted as “easy” if it is the most commonly found form under most crystallisation conditions. Here, we calculate the difference in normalised rugosities between the polymorph that is easy to crystallise and the polymorph that is hard to crystallise, ${\Delta \bar{R}}_{depth}^{N}\left[eh\right]=\;{\bar{R}}_{depth}^{N}\left(easy\right)-{\bar{R}}_{depth}^{N}\left(hard\right)$ . For those pairs in Table 1 having |${\Delta \bar{R}}_{depth}^{N}\left[eh\right]|<0.04\;$ we see no significant links between the crystal rugosity and the ease of crystallisation. However, when the crystal rugosity differences become significant (${|\Delta \bar{R}}_{depth}^{N}\left[eh\right]|>0.04$ ), the form with the rougher surfaces is usually also the form which is harder to crystallise (thus ${\Delta \bar{R}}_{depth}^{N}\left[eh\right]$ is positive). In examining the link between rugosity and polymorph stability it is noted that these are not necessarily correlated. However, when the rougher crystal form is also the most stable, thermodynamics favours this form but kinetics does not. These are the cases of theophylline‐IV^[26]^ and ritonavir‐II.^[27]^ These two forms, despite being the most stable, took a long time to discover experimentally. We find the case of theophylline form IV especially fascinating. Even though theophylline has been crystallised for many years, form IV was only reported for the first time in 2011. A detailed study of this form by Bobrovs et al.^[26]^ concluded that it is very difficult to nucleate. Only by slurrying the more common form II in well‐dried solvents for several months did form IV start appearing. The considerably rougher set of surfaces of the stable theophylline‐IV may be responsible for its difficulty of nucleation (Figure 2). Theophylline‐II, however, has smooth surfaces and the form can be obtained readily from most solvents. When the rougher polymorph is also the metastable form both thermodynamics and kinetics are against its appearance as seen for aspirin‐II,[^18^, ^19^] curcumin‐II,^[20]^ paracetamol‐II,^[24]^ carbamazepine‐V^[25]^ and 5‐Br‐aspirin‐II.[^22^, ^23^] In all these cases, the forms were only discovered in the presence of impurities which may act by inhibiting the growth of the stable form.^[28]^

**Figure 2:**
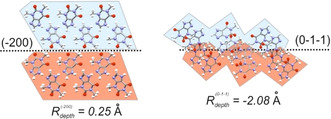
Illustration of the differences in rugosity between the most dominant face of form II (left) and form IV (right) of theophylline.

Next, we looked at a number of notorious polymorphic systems with many known forms (ESI). When experimental data on the stability of forms was available (e.g. ROY,^[29]^ glycine^[30]^), we noticed that highly metastable forms which crystallised readily tended to be smooth polymorphs. For example, in ROY, the more metastable YN (${\bar{R}}_{depth}^{N}=-0.057$ ) and R (${\bar{R}}_{depth}^{N}=-0.102$ ) forms were smoother than the stable room temperature Y form (${\bar{R}}_{depth}^{N}=-0.265$ ).^[29]^ In the case of glycine,^[30]^ the metastable α (${\bar{R}}_{depth}^{N}=-0.081$ ) crystallises preferentially from aqueous solutions, while the stable *γ* (${\bar{R}}_{depth}^{N}=-0.087$ ) can be obtained only by using tailor‐made additives or working at a pH different from the isoelectric point. β‐Glycine has the lowest rugosity (${\bar{R}}_{depth}^{N}=-0.039$ ) and it quickly transforms to α but there is no information as to how often it nucleates before α.

Finally, it has not escaped our attention that this normalised crystal rugosity model may have a significant application in crystal structure prediction (CSP). This idea flows naturally from our previous work on nucleation and growth of benzoic acids which demonstrated the link between nucleation and growth rates.^[31]^ Here we take two specific examples of CSP outputs, one for diflunisal and a second for the proprietary compound X. In Figure 3 we represent the landscape as relative lattice energy (Δ*E*_lattice_) of each crystal structure versus its normalised average crystal rugosity. The vertical lines bracket the 98 % attainable space between the smoothest polymorph in the landscape within 4 kJ mol^−1^ of the global minimum (typical of polymorphs) and the rougher polymorph possible with −0.2 rugosity from the smoothest form (this limit is adopted based on our CSD statistics from Figure 1). For diflunisal, Figure 3 a, two ordered crystal structures in the landscape (Ia, Ib) are components of the experimental disordered form I. These two structures are amongst the smoothest forms in the low energy region. This matches well with the fact that form I crystallises readily from solutions. Form III is predicted as the global minimum but with a higher roughness than form I. Experimentally this is manifested as form III being harder^[32]^ to nucleate and usually obtained via a solution‐mediated transformation (as with theophylline‐IV). For compound X, with two known polymorphs, a complex landscape is produced as seen in Figure 3 b. Polymorph I is predicted to be amongst the smoothest forms in the landscape. This marries well with the fact that form I readily crystallises from solution. Form II is enantiotropically related to I and can only be obtained at low temperatures. We might then ask the question as to whether it is possible to isolate the most stable predicted form for compound X having a crystal rugosity of around −0.34. This predicted most stable form (at nominal 0 K) lies above typical roughness differences in polymorphs in the CSD (Figure 1) and in the notorious polymorphic cases analysed in Table 1, hence we expect that its observation is experimentally hindered and indeed, it has never been isolated to date. We note that the ${\Delta \bar{R}}_{depth}^{N}\;$ cut‐off value of −0.2 used to assess whether the computationally generated polymorphs are plausible, is relative to the smoothest predicted form in the low energy version of the landscape and thus would be shifted in absolute terms depending on the compound and its landscape.

**Figure 3:**
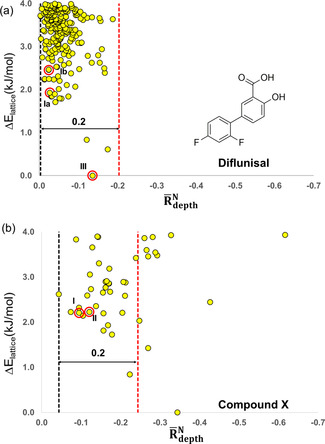
CSP landscapes: a) diflunisal and b) compound X. Experimental structures are circled in red.

In conclusion, we have derived a model to compute normalised crystal surface rugosities in molecular crystals based on the roughness model of Bryant, Maloney and Sykes.^[11]^ Given the possible link between the ease of crystallisation and roughness of crystal surfaces as revealed by two previous studies, we have computed rugosity differences in polymorphs in the CSD. It is concluded that for 98 % of polymorphic families the difference in normalised rugosity between the smoothest and roughest structures does not exceed 0.2. The utility of this concept has been tested by examining some notorious systems with forms which are hard to produce. We have found good correlations between our calculated rugosities for these materials and their ease of crystallisation. In applying this idea to CSP lattice energy landscapes we find that, by using the roughness parameter, ${\Delta \bar{R}}_{depth}^{N}$ as a measure of crystallisability combined with the computed lattice energies and the fact that in most polymorphic systems ${\Delta \bar{R}}_{depth}^{N}$ does not exceed 0.2, it is possible to identify those forms most readily accessible through experimentation. We do not expect this model to be infallible for several reasons: i) conformational aspects of crystallisation are important and also need accounting for, ii) some rough surfaces may be stabilised in solution due to favourable solvent–surface interactions, iii) the presence of small levels of impurities in the solution together with other more complex nucleation pathways may play a role in the observation of forms and iv) the method presented here is based on a simplified 1D description of rugosity and BFDH morphologies which make no account for solvent effects. Whilst we are now working to improve our method of crystal rugosity calculations, this novel concept and the method presented here can already be applied as a quantifiable tool bringing a new interpretation of CSP lattice energy versus crystallisability landscapes. Given how ubiquitous these landscapes have become in solid form development of pharmaceuticals, this method should be of interest to the community.

## Conflict of interest

The authors declare no conflict of interest.

## Supporting information

As a service to our authors and readers, this journal provides supporting information supplied by the authors. Such materials are peer reviewed and may be re‐organized for online delivery, but are not copy‐edited or typeset. Technical support issues arising from supporting information (other than missing files) should be addressed to the authors.

Supplementary
