# Supplementary material for: Transforming Computed Energy Landscapes into Experimental Realities: The Role of Structural Rugosity
Source: Angew Chem Int Ed Engl. 2020 Sep 2;59(46):20357–60. doi: 10.1002/anie.202006939 (PMC7693212; doi:10.1002/anie.202006939)
Supplement: Supplementary file 1 — Supplementary [file ANIE-59-20357-s001.pdf]

Angewandte Chemie
Eine Zeitschrift der Gesellschaft Deutscher Chemiker

Supporting Information

Transforming Computed Energy Landscapes into Experimental Realities: The Role of Structural Rugosity

*Riccardo Montis, Roger J. Davey, Sarah E. Wright, Grahame R. Woollam, and Aurora J. Cruz-Cabeza**

anie_202006939_sm_miscellaneous_information.pdf

S1. Particle Rugosity Method **2**
S2. Polymorphic Dataset and rugosity histograms **4**
S3. Polymorphic systems which are easy vs. hard to crystallise **5**
S3.1. Aspirin 6
S3.2. Dapsone 6
S3.3. Axitinib 7
S3.4. *p*ABA 7
S3.5. 5-BrAspirin 8
S3.6. Carbamazepine 8
S3.7. Paracetamol 9
S3.8. Rotigotine 10
S3.9. Curcumin 10
S3.10. Theophylline 11
S3.11. Ritonavir 11
S4. Notorious Polymorphic Systems **12**
S4.1. Glycine 12
S4.2. Acridine 13
S4.3. Coumarin 13
S4.4. ROY 14
S5. CSP Methods **15**
S6. References **15**

S1. Particle Rugosity Method

The algorithm used to compute the normalise crystal rugosities is broken in four steps in figure S1. This algorithm was coded in python making use of the functionalities available in the CSD python API.

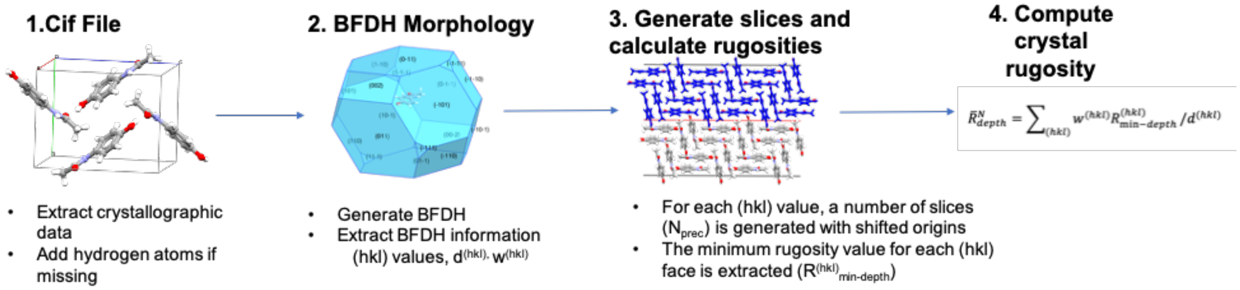

Figure S1. Steps coded into the crystal rugosity algorithm.

Step 1. The crystallographic information of the structure of interest (with a given Refcode) is retrieved from the CSD (or can be loaded via a cif file). If the molecule has missing hydrogens, these are added using the standard procedure implemented in the CSD Mercury.

Step 2. The BFDH morphology is generated from the crystallographic information of the structure of interest using the python API with its standard settings. With this, the (hkl) values of the important BFDH faces are extracted as well as the BFDH areas for each (hkl). The morphological importance of each (hkl) face is then calculated as $w^{(hkl)}=Area^{(hkl)}/Area^{BFDH}$ where $Area^{(hkl)}$ is the area of an individual (hkl) face and $Area^{BFDH}$ is the total area of the BFDH morphology. The d-spacings, $d^{(hkl)}$, for those faces are then computed using the method implemented in the CSD python API. If possible, faces where grouped into families making use of crystal symmetry to reduce the number of rugosity calculations since faces of the same family {hkl} will have identical topologies and rugosities.

Step 3. For each of the relevant BFDH (hkl) faces, the minimum value of rugosity was calculated in the following way.

- A number of slices (N_{prec}) for the same (hkl) face were generated. The original slice was generated at the (hkl) plane. Following slices were generated by shifting the origin of the (hkl) slice by (d_{hkl}/N), N_{prec} times where N is 2, 3,…, N_{prec}. For example, for a (001) plane with $d^{(001)}$=10 Å, and a N_{prec}=4, four slices are generated:
    - Slice with no origin shift
    - Slice with origin shift by 2.5 Å (10/4)
    - Slice with origin shift by 3.3 Å (10/3)
    - Slice with origin shifted by 5.0 Å (10/2)

- The Bryant, Maloney and Sykes[1] method was used to generate two consecutive slices. A slicing thickness of 14 Å was employed (the standard is 10 Å but this was found to be too small for some large molecules) together with a width of 30 Å and a number of shifts (see above), and the $R^{(hkl)}_{depth}$ for each slice (and shift) calculated.
- For each (hkl), the computed rugosities at the different shifts were then compared and the minimum value (in absolute terms) was then taken as the true value of the rugosity parameter for that specific (hkl) face. We refer to this parameter as $R^{(hkl)}_{\min-depth}$.
- An example of the impact of the shift is given in Figure S2 for Paracetamol form I, with refcode HXACAN07. The smoothest surface (in this case the face cut at the origin) would be preferably shown in the crystal morphology since it is most likely to have the lowest surface energy thus be displayed in the morphology.

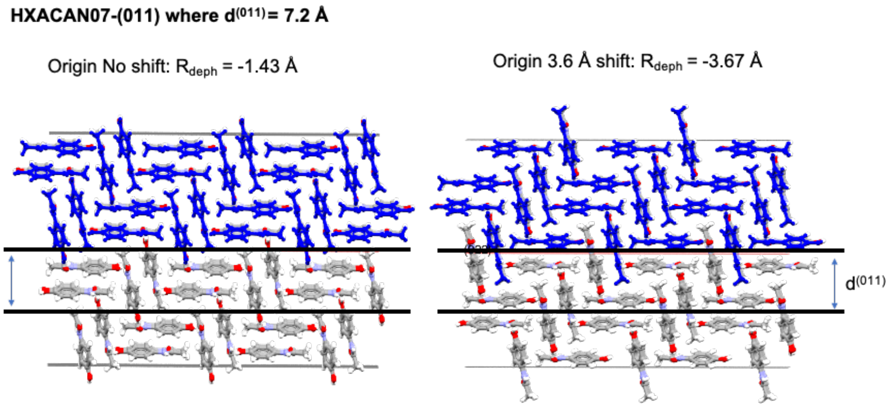

Figure S2. Impact of the slice origin shift for the (011) face in paracetamol form I (HXACAN07). In this case, the -1.43 Å is taken as the value for the (011) face since it is the smoothest surface. The slice of interest is limited by the black line. The layer of molecules in the slice above are coloured in blue to illustrate the degree of molecular interpenetration.

Step 4. The normalised crystal rugosity $\bar{R}^N_{depth}$ is then calculated as,

$$\bar{R}^N_{depth} = \sum\nolimits_{(hkl)} w^{(hkl)} R^{(hkl)}_{\min-depth} / d^{(hkl)}$$

To illustrate the impact of the number of shifts explored in the computation of the rugosities, the overall $\bar{R}^N_{depth}$ was calculated as a function of the number of shifts (N_{prec}) for polymorphs I and II of paracetamol and polymorphs II and IV of theophylline (see Figure S3). The value of N_{prec}=4 was found to be a good compromise between accuracy of the computed rugosity and the required computational time.

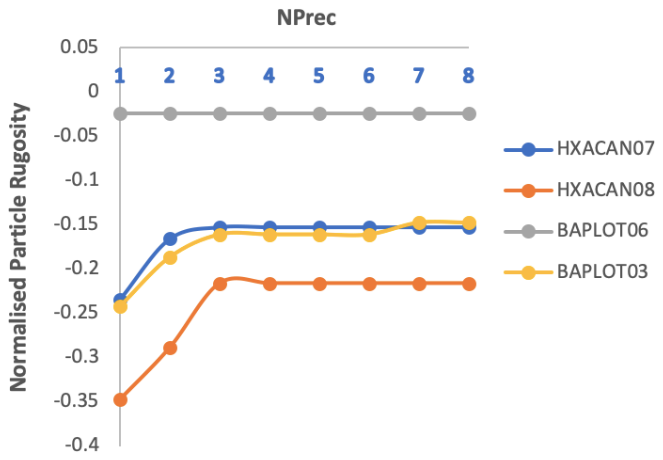

Figure S4. Impact of N_{Prec} on the calculated particle rugosity for paracetamol forms I & II (HXACAN07, HXACAN08) and theophylline forms II & IV (BAPLOT06, BAPLOT03).

S2. Polymorphic Dataset and rugosity histograms

Conquest was used to retrieve crystal structures of organic compounds from the CSD[2] (version 4.39 Nov 2017) containing only one chemical component and the most common elements in molecular crystals (H, D, C, O, N and halogens). This resulted in 187 895 crystal structures. A python script was then written making the use of the CSD python API to analyse these structures. With the script, each refcode family was analysed and all crystal structures in each refcode family were compared using the COMPACK algorithm[3] (with the standard settings and a clusters of 20 molecules). This allowed for removal of crystal structure redeterminations and for the splitting of the dataset in a Monomorphic and Polymorphic dataset. The polymorphic dataset taken forward for rugosity calculations contained 5611 crystal structures belonging to 2559 refcode families. Of those 5611 crystal structures, we note that only 198 of them were solved from Powder Solution methods whilst the overwhelming majority were solved from single crystal X-Ray diffraction methods (5413 structures).

Figure S5 shows the distribution of normalised rugosities for all crystal structures (left) and the maximum rugosity difference between polymorphic families (right).

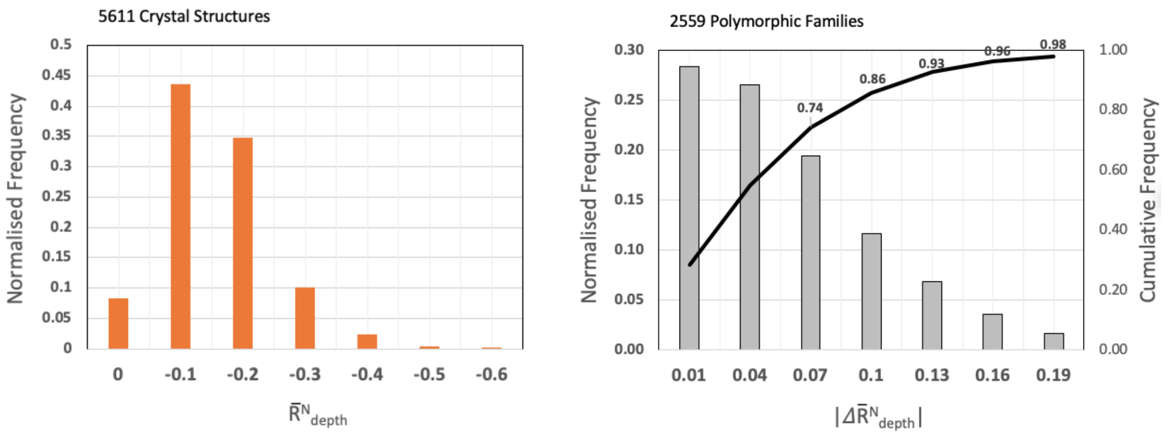

Figure S5 Distribution of normalised rugosities for all crystal structures (left) and the maximum rugosity difference between polymorphic families (right).

S3. Polymorphic systems which are easy vs. hard to crystallise

We defined a list of pair of polymorphs that are easy or difficult to crystallise. The choice of the category "easy" or "difficult" is made on the basis of the experimental data availability in the literature. We define "difficult", any compound for which a direct crystallisation from solution is not straightforward. This definition might encompass all those polymorphic forms that can be only obtained by solid-solid transformations, solvent-mediated transformations or simply by solvent removal from a solvate form. Other examples might be systems that can be crystallised only using a specific solvent or polymorphic forms promoted by impurities or particular conditions (late appearing polymorphs). In the following paragraphs we summarise previous experimental results for each compound reported in table S1.

Table S1. Average normalised crystal rugosities for notorious polymorphic systems with a form hard to produce and a more common form easier to crystallise (N_{prec}=4).

	**Easy**			**Hard**			
**Compound**	**REFCODE**	**Form**	$\bar{R}^N_{depth}$(Å)	**REFCODE**	**Form**	$\bar{R}^N_{depth}$(Å)	$\Delta\bar{R}^N_{depth}$(Å)
Rotigotine§	RALMOG	I	-0.402	RALMOG01	II*	-0.372	-0.030
Dapsone	DAPSUO14	III	-0.109	CIF[4]	V*	-0.106	-0.003
*p*ABA	AMBNAC07	α	-0.061	AMBNAC08	β**	-0.091	0.030
Aspirin	ACSALA01	I	-0.070	ACSALA15	II	-0.121	0.051
Curcumin§	BINMEQ	I*	-0.233	BINMEQ08	II	-0.288	0.055
Axitinib§	VUSDIX	XXV	-0.115	VUSDIX04	XLI*#	-0.170	0.055
5-BrAspirin	NUWTIJ	I	-0.061	NUWTIJ01	II	-0.117	0.056
Paracetamol	HXACAN07	I*	-0.153	HXACAN08	II	-0.216	0.063
Carbamazepine	CBMZPN10	III*	-0.125	CBMZPN16	V	-0.193	0.068
Theophylline	BAPLOT06	II	-0.025	BAPLOT03	IV*	-0.161	0.136
Ritonavir§	YIGPIO02	I	-0.114	YIGPIO03	II*	-0.265	0.151

*Thermodynamically stable form at ambient temperature conditions. **Thermodynamically stable at low T. §Conformational polymorphs. #Via conversion from a solvate.

S3.1. Aspirin

Aspirin (acetylsalicylic acid) is perhaps one of the most popular nonsteroidal anti-inflammatory drugs. It is used in the treatment of inflammation, pain, fever and, at low doses, to prevent different heart conditions. It was synthetised more than 150 years ago to improve taste, gastric tolerability and pharmacological properties of salicylic acid. Aspirin was for long time assumed to exist only as one crystalline form (Form I), though, during the 70s, some studies based on the observation of differences in melting points, dissolution and crystal habits, suggested the possibility that aspirin could show polymorphism. With the development of CSP methods, polymorphism of aspirin became a target of different studies. In 1995 Gavezzotti[5] proposed a tentative polymorph using a structure-construction procedure. Few years later, Payne et al.[6] showed that a planar conformation of aspirin could be plausible and suggested that experimental conditions promoting the stability of this conformer could lead to new polymorphs. In 2004, Ouvrard and Price[7] predicted the existence of a new polymorph of aspirin showing a crystal packing very similar to form I. This form was experimentally isolated by Vishweshwar *et al.* in 2005.[8] Form II only differs to form I by a small shift of adjacent layers. Later, Bond et al.[9] proved that the structure reported for form II consisted of an inter-grown phase. Recently, Bond proved that pure form II can be prepared in the presence of impurities of aspirin anhydrate.[10] Recently a third form of aspirin (form III) has been discovered at high pressure;[11] however, its crystal structure was not determined. This form transforms to form I upon release of pressure. A third ambient Aspirin polymorph[12] was very recently discovered from the melt and the crystal structure determined (form IV). Form IV transforms form I at room temperature.

Only form I can be crystallised with conventional methods; form II can be obtained in its pure form only seeding solutions of aspirin with 15% wt of its anhydrate. The remaining two polymorphs can only be obtained at high pressure and from the melt respectively. The fact that form I is easier to crystallise than form II, seems to be consistent with its smoother nature ($\bar{R}^{N}_{depth}$ [I] = -0.070, $\bar{R}^{N}_{depth}$ [II] = -0.121). However, we note here that the structures of the crystals and the surfaces are so similar on so are the rugosities.

S3.2. Dapsone

Dapsone (4,4’-diaminodiphenyl sulfone) is an API used in the treatment of leprosy in combination with rifampicin and clofazimine. Dapsone is known to crystallise as five polymorphic forms (forms I-V) and solvates including an unusual non-stechiometric hydrate that by de-solvation produces an isomorphic dehydrate (Hy_{dehy}).[13–16] The stability order of I-V, determined on the basis of lattice energy calculations and confirmed by calorimetric measurements, is the following: V (most stable) > III > Hy_{dehy} > II > I > IV (least stable). Form V is a late appearing polymorph recently discovered[4] and corresponds to the thermodynamically stable form in the range absolute zero-90 °C. Above 90 °C form II is more stable and, at higher temperatures, form I becomes the most stable. Polymorphs II/III, III/I and II/I are enantiotropically related.[4] Form I is the high temperature form, it can be obtained by annealing the melt at temperatures above 170 °C. Desolvation of solvate forms below and above 75 °C results in form III and form II respectively. In both cases, the product also presents some impurities of form I. Crystallisations by slow evaporation or slow cooling from solvents, excluding those forming solvates, produce form III. Form

IV cannot be crystallised form solvents but can be produced concomitantly with form III, by annealing quench-cooled melts of dapsone at 50-70 °C. Form V can be obtained form by slurring any of the polymorphic forms in non-solvating solvents, in the range of temperatures 10-90 °C.

Before the discovery of the late appearing form V, form III was indicated as the thermodynamically stable form at room temperature.[17] Form III shows indeed a remarkable kinetic stability and proved to be stable at ambient conditions for approximately 40 years. This, together with the slow nucleation and growth rate of form V, delayed the discovery of the late appearing polymorph V.[4] Although from V proved to be difficult to crystallise when compared to III, the values of rugosity of both forms (table S1) were found to be very similar ($\bar{R}^{N}_{depth}$ [III] = -0.109, $\bar{R}^{N}_{depth}$ [V] = -0.106).

S3.3. Axitinib

Axitinib is an API developed by Pfizer for the treatment of carcinomas. Polymorphism of axitinib has been extensively investigated both experimentally[18,19] and computationally[20,21]. To date, 71 different forms including anhydrous, solvates and hydrates have been reported in the literature[19]. Five polymorphic forms of axitinib have been discovered (I, IV, VI, XXV and XLI)[18,19]. XLI is the most stable form and is monotropically related to the other polymorphs. IV, VI and XXV are enantiotropically related. Form I is the least stable form and transforms into the monohydrate IX at high relative humidity. On the basis of Differential Scanning Calorimetry data, solubility measurements and Burger's rule, the order of stability is the following: XLI > XXV ≈ VI > IV > I. This order does not correlate with the calculated densities. This discrepancy has been ascribed to the conformational flexibility of axitinib[19].

Due to the high propensity of axitinib to form solvates, the isolation of anhydrous phases is difficult. Standard crystallisations mostly produce solvates. In the majority of these solvates, desolvation temperatures are higher than the boiling point of the solvent. Desolvation of solvates produces form IV and form XXV. The most stable form XLI can be obtained only applying a targeted procedure consisting of slurring solvates at high temperatures[19].

In the case of Axitinib none of the polymorphs can be nucleated directly from solution. In this respect, polymorphs of Axitinib should be all classified as "difficult" to crystallise. The measured rugosity for Axitinib polymorphs shows that the thermodynamically stable form XLI is less smooth when compared to XXV ($\bar{R}^{N}_{depth}$ [XLI] = -0.170, $\bar{R}^{N}_{depth}$ [XXV] = -0.115) consistency with the difficulty of its nucleation.

S3.4. *p*ABA

para-aminobenzoic acid *(p*ABA) represents a popular model compound for solid state studies. Previous work on *p*ABA covers several aspects of organic crystalline materials field, such as solubility measurements, nucleation and growth kinetics, crystal morphology,[22,23] polymorphism[24,25] and co-crystals.[26,27] In particular, polymorphism of *p*ABA has been extensively investigated in recent years. *p*ABA is known to crystallise as four polymorphic forms. Two of them, α and β are very well known and have been the object of most of studies cited above. Recently, two new forms γ[28] and δ[29] have been isolated by crystallisation in the presence of impurities and at high pressures, respectively. α and β are enantiotropically related, with a transition temperature at 13.8 ˚C. β is the most stable form below this

temperature, at higher temperatures, α becomes the most stable. Previous studies show that on heating, β transforms endothermically to α in the range of temperatures 70-100 ˚C. At high pressures, α transforms to δ. More details on the four polymorphs, including descriptions of morphology, kinetic of nucleation and growth, crystal structures and crystal structure prediction are also summarised in a recent paper by Cruz-Cabeza and Davey.[24]

Differently to α, that can be easily crystallised from several solvents and supersaturations,[25] β can be only obtained at specific experimental conditions. Previous studies suggest that β can be consistently obtained from water at supersaturations in the range 1.1-148. Alternative routes, involve slurring α below 14 ˚C or by controlled sonication.[30] A recent work by Nagy[31] shows that the application of a semibatch supersaturation control (SSC) strategy for cooling crystallisation consistently produced β- *p*ABA.

Like the previous cases, α and β-*p*ABA represent a suitable model system for pair of polymorphs that, on the basis of the criteria described above, can be considered as easy and difficult to crystallise. This is consistent with the observation that α is smoother than β ($\bar{R}^N_{depth}$ [α] = -0.061, $\bar{R}^N_{depth}$ [β] = -0.091).

S3.5. 5-BrAspirin

Differently to the parent molecule aspirin, there is no much information on 5-bromoaspirin (5-BrAsp) in the literature. The crystal structure of 5-BrAsp (form I) was reported for the first time by Hursthouse et al.[32] as a part of a larger study on the systematic structural comparison of several substituted derivatives of aspirin. Screening from different solvents always produced the known form I. A new polymorph (form II) was reported by Bond et al.[33] in a study on the influence of impurities in promoting polymorphism. Crystallisations from common solvents always produce form I. Form II can only be obtained in the presence of impurities of 5-bromoaspirin anhydrate. Slurring form II in organic solvents always produces form I as the result of a solvent-mediated transformation and indicating that, in the range temperatures used for these experiments, form I is the thermodynamic form.[33] The fact that form II requires non-convetional procedures to be isolated seems to be consistent with its rougher nature when compared to form I ($\bar{R}^N_{depth}$ [I] = -0.061, $\bar{R}^N_{depth}$ [II] = -0.117).

S3.6. Carbamazepine

Carbamazepine represents another popular model compound for polymorphism studies. It is used as antiepileptic drug and as analgesic for the treatment of trigeminal neuralgia. Over 50 different solid forms of carbamazepine have been reported so far.[34,35] These include several solvates, hydrates, co-crystals[36,37] and five polymorphic forms (form I-V).[35,38–41]

Much of the work reported in the literature is focused on forms I-IV. Form V was only recently discovered[35] and differs to I-IV for the absence of the centrosymmetric dimer, which is a robust structural feature in CBZ crystal structures. Form V represents indeed the first example of polymorph of carbamazepine showing catemeric supramolecular arrangements and, accordingly, it was experimentally

isolated applying a computationally assisted strategy, and using the catemeric structure of dihydrocarbamazepine as template.

Forms I-IV are often named according to their crystalline system as follow: form I (triclinic), form II (trigonal), form III (p-monoclinic) and form IV (c-monoclinic). Previous analysis based on density and thermochemical data suggests that form III and form I are enantiotropically related, with form III the thermodynamic stable form from room temperature to 70 °C. Form III is also related enantiotropically to form IV. There is no agreement on the relationship occurring between form IV and form I. Grezsiak et al.[38] described this pair as enantiotropically related. Mielck[42] suggests they are monotropically related. As pointed by Harris et al.[43] the fast transformation of form I does not allow the determination of its melting point, making difficult to identify the thermodynamic relationship between this polymorphs and the others. Forms I-IV are close in energy and have the following order of stability at room temperature: III < I < IV < II. The small differences in energy suggest that the choice of experimental conditions is very important for the polymorphism outcome. Previous works confirm that, in the case of CBZ, the choice of supersaturations, stirring and solvents represent important factors for both, the nucleation of specific polymorphic forms and solvent-mediated transformations.[44]

Among the five polymorphs of CBZ, we selected form III and form V as examples of easy and difficult to crystallise polymorphs. According to the literature, form III easily crystallises from most organic solvents ($\bar{R}^N_{depth}$[III] = -0.125).,[45] while form V[35] is the only polymorph that requires a specific protocol involving seeds of the templating dihydrocarbamazepine ($\bar{R}^N_{depth}$ [V] = -0.193).

S3.7. Paracetamol

Paracetamol (also known as acetaminophen or tylenol), represents one of the most widely used antipyretic and analgesic drugs. It exists as different polymorphic forms and related forms.[46] Among these, only crystal structures of four polymorphic forms have been solved and refined so far: form I (monoclinic),[47] form II (orthorhombic),[48] III (orthorhombic)[49] and the recently discovered form III-m (monoclinic),[50] obtained from powder diffraction data and later renamed as form VI.[51] Recent studies have also suggested the existence of two high-pressure polymorphs (IV and V) [52] and three more ambient polymorphs (VII, VIII and IX), obtained from the melt.[51] Form I and form II have been extensively investigated and characterised. Form I is the most stable polymorph under ambient conditions, it can be easily crystallised from different solvents. It shows poor compressibility, due to the absence of slip planes in its crystal structure and consequently it requires the uses of additives for tablet preparation. Form II shows better compressibility and higher solubility in water; however, it is metastable and tends to transforms to form I.[53] Furthermore, crystallisation of form II from solution is difficult. It was first crystallised by slow evaporation from ethanol. Recent attempts to obtain form II with this procedure were unsuccessful. A high-throughput polymorphism study by Peterson et al.[54] investigated the polymorphism of paracetamol by thermal cycling in different solvents and at different supersaturations, obtaining form II only using specific experimental conditions. Recent attempts to produce form II involved the use of different procedures. A summary of them is reported in a recent review by Cruz et al.[55] The comparison

of the measured rugosity for form I and II shows that form I is smoother than form II ($\bar{R}^N_{depth}$ [I] = -0.153, $\bar{R}^N_{depth}$ [II] = -0.216).

S3.8. Rotigotine

Rigotigone is a dopamine agonist known since the 80s and now used in the treatment of Parkinson's disease and restless legs syndrome.[56] It crystallises as two conformational polymorphs, form I and II respectively. Form I was the only known form until a more stable polymorph, form II, was discovered. As in the case of ritonavir (see below), the discovery of this late appearing polymorph, caused in 2008 the withdrawal from the market of the original formulation.[57] The discovery of form II was not expected, no evidence of this more stable form was observed in the pre-formulation screening. Low temperatures limit the growth of form II. However, as shown by Rietveld et al.[57] form I is monotropic and metastable with respect form II in the whole temperature-pressure domain. A recent CSP study predicted a third polymorph of rigotigone lying between form I and form II.[58]

Form I can be considered as "easy" to crystallise ($\bar{R}^N_{depth}$ I = -0.402). Conversely, the late appearance of the most stable form II can be seen as an indication of a phase "difficult" to crystallise ($\bar{R}^N_{depth}$ II = -0.372). In this case, the values of the rugosities are reversed with the form harder to crystallise being smoother.

S3.9. Curcumin

Curcumin is major constituent of the turmeric spice. It is a poorly water-soluble compound with anti-microbial, anti-inflammatory and anti-oxidant properties and potential clinical efficacy towards several diseases.[59,60] Several aspects of the solid state properties of curcumin have been investigated, a summary of these results can be found in a recent paper by Suresh and Nangia.[59]

To date, three polymorphic forms,[61,62] four co-crystals[63] and one chloroform solvate[64] of curcumin have been isolated. The first structure (form I) has been solved in 1982 by Tonnesen et al.[61] and corresponds to a monoclinic (*P*2/n) structure. More recently two new orthorhombic polymorphic forms (form II: $Pca2_1$; from III: *Pbca* respectively) have been isolated by Sanphui et al.[62] In all the structures, the molecule exists in the b-keto–enol tautomer form. Form II and III adopt a planar conformation, while in the case of form I this is slightly twisted. Accordingly, the three polymorphs of curcumin have been indicated as a case of tautomer and conformational polymorphs.[59]

Form I is the most stable polymorph, form II and III transform to I before melting. The stability order is the following: I > II > III.[62] Form I can be easily crystallised at room temperature from several common solvents. This is consistent with the fact that its measured rugosity is lower when compared, for example, to form II ($\bar{R}^N_{depth}$ [I] = -0.233, $\bar{R}^N_{depth}$ [II] = -0.288). Crystallisation of form II and III is indeed more difficult. They were initially obtained as the result of cocrystallisation in the presence of 4-hydroxypyridine in EtOH. Further studies[65–67] suggest that crystallisation of form II and III can be induced by using ultrasounds and specific additives.

S3.10. Theophylline

Theophylline is a methylxanthine used for the treatment of asthma. It exists as four polymorphic forms (I- IV), a monohydrate, a DMSO solvate and several cocrystals.[68–73] Theophylline is a widely studied model system in solid state chemistry. Most of the work previously reported, focus on hydration-dehydration behaviour and cocrystal formation. There are also some works focusing on the polymorphism of theophylline and on the characterization, structural determination crystallisation and stability of its polymorphic forms.[74,75] To date, only crystal structures for form I,[76] form II,[77] form IV[76] have been determined. Until the discovery of form IV, Form II was for long time considered as the thermodynamically stable form. Form II, is metastable and is enantiotropically related to form I. It undergoes transformation to this form at about 232 ˚C. All the polymorphic forms of theophylline convert to the monohydrate M in contact with water or at high relative humidity.[74,78] Dehydration of the monohydrate produces either form II or III; form III then transforms to form II. The stability order for these forms has the following order: Form IV > Form II > Form I; form III is highly metastable.[76]

Form II is the kinetic product of crystallisation. All the transformations relating the known polymorphs of theophylline are mediated by form II. Form II can be easily crystallised from non-aqueous solutions at room temperature. Form IV is the thermodynamically stable form; it can be obtained only by solvent mediated transformations. Its late discovery was recently explained on the basis of thermodynamic and structural features.[76]
In the context of our study, form IV represents a system "difficult" to crystallise whereas crystallisation of form II is "easy " ($\bar{R}^{N}_{depth}$ II = -0.025, $\bar{R}^{N}_{depth}$ IV = -0.161).

S3.11. Ritonavir

Ritonavir represents one of the most famous examples of disappearing polymorph.[79,80] It was developed at Abbott laboratories in 1992 as protease inhibitor for HIV and marketed as a semi-solid capsule formulation. Few years after the introduction into the market, due to the appearance of the more stable polymorph II, not observed during the development and manufacturing steps, some lots failed solubility tests and the product was removed from the market.[81,82] The new form II showed a significantly lower solubility and a different molecular conformation.[83] After the discovery of form II, the crystallisation of form I was difficult becoming a case of disappearing polymorph. Form I crystallises as lath-shaped crystals and the molecule adopts a trans conformation around the carbamate torsion angle. Form II adopts the less favourable cis configuration and crystallises as needle-shaped crystals. Form II is the most stable and has higher melting point and heat of fusion if compared to form I.[79] Although form II is the thermodynamically stable form, its crystallisation is more difficult and requires the presence of seeds, impurities or high supersaturations, due to the fact that in order to nucleate it requires the formation of the less stable cis-isomer. In particular, the first appearance of form II was ascribed to a degradation of ritonavir into a cyclic carbamate product, able to template the crystallisation of the *cis*-isomer.[82] Accordingly, form II represents a case of polymorph difficult to crystallise, being not observed during development and manufacturing steps and resulting in a late appearing polymorph. Except in the case of the presence of seeds of form II, crystallisation of form I is initially favoured, classifying this form as easier to crystallise than form II ($\bar{R}^{N}_{depth}$ [I] = -0.114, $\bar{R}^{N}_{depth}$ [II] = -0.265). Beyond the surface rugosity difference,

here the key issue is also the conformational change required. Form II has a higher metastable conformer and this is also linked to its difficulty of nucleation and growth.

A more recent high-throughput polymorphism screening identified a new metastable polymorph (form III), one hydrate form and a solvate.[84]

S4. Notorious Polymorphic Systems

S4.1. Glycine

Glycine is known to crystallise as three ambient polymorphs α-, β- and γ-glycine.[85–87] Recently, other three forms (named δ, ε and ξ respectively) have been obtained at high pressure.[88] For the purposes of this work, we limit our discussion only on α-, β- and γ-glycine. The three ambient polymorphs of glycine crystallise all in the zwitterionic form; α-glycine packs as centrosymmetric dimers, while β- and γ-glycine adopt non-centric arrangements. The stability order at ambient conditions is as follow: γ (more stable) > α > β (least stable).[89] β-glycine is unstable, it rapidly transforms to the α polymorph in the presence of humidity or in aqueous solutions.[86] On the contrary, the solvent-mediated transformation of α-glycine into the γ polymorph is slow. α-glycine can be easily crystallised from aqueous solutions while β-glycine requires the presence of water-alcohol solutions.[90,91] Crystallisation of γ-glycine from aqueous solutions is difficult and it can be obtained by using tailor-made additives or working at pH different than the isoelectric point [92,93] crystallisations at the natural isoelectric pH (5.97) always produce α, despite the higher stability of γ.[94] A recent work by Vesga et al.[95] shows that γ-glycine can be obtained at in the absence of stirring from highly supersaturated aqueous solutions and suggests that stirring might result in a preferential crystallisation of α-glycine. However, in most of the previous studies α-glycine proved to be easy to crystallise while the γ polymorph, generally requires specific conditions for its crystallisation, so it can be defined as difficult to crystallise.[96]

Table S2. Calculated $\bar{R}_{depth}^{N}(\text{Å})$ for α-, β- and γ-glycine.

Glycine Stability order*	REFCODE	Form	$\bar{R}_{depth}^{N}(\text{Å})$
#3	GLYCIN81	β	-0.0389
#2	GLYCIN99	α	-0.0811
#1	GLYCIN82	γ	-0.0873

*1 being the most stable and 3 the least stable.

Calculated rugosity values show that the β and α are smoother than γ (see Table S2), consistent with the easiness of crystallisation. The β despite being the least stable, has the smoothest surface which might favour the kinetics for its nucleation and observation. The γ polymorph, on the contrary, is the most stable but also the one hardest to crystallise (with the rougher surfaces).

S4.2. Acridine

Acridine is known to form eight polymorphic forms and one hydrate (form I). Experimental work published over the last few years is summarised in a recent review by Stephens et al.[97] In particular, results from previous works shows that the eight polymorphs of acridine can be often obtained applying similar or identical experimental conditions. In some cases, same crystallisation conditions lead to mixtures of concomitant polymorphs. Form II, III and IV have been all prepared by sublimation under vacuum[98] and by evaporation from several solvents.[99–101] Form IV was also prepared concomitant with VII by evaporation in the presence of terephthalic acid.[97] Values of calculated rugosity show that II and III are smoother than IV ($\bar{R}^N_{depth}$[II]= -0.076, $\bar{R}^N_{depth}$[III]= -0.117 and $\bar{R}^N_{depth}$[IV]=-0.174).

Forms VI and VII have been discovered during a study of the influence of dicarboxylic acids in solution during crystallisation of acridine,[99] suggesting that the isolation of these two forms might require specific experimental conditions ($\bar{R}^N_{depth}$[VI]= -0.158 and $\bar{R}^N_{depth}$[VII]=-0.160). However, other studies showed that VI and VII can be also obtained by quenching molten acridine, form VII was also obtained by crash-cooling acetone solutions.[101]. Form IX was only discovered recently and crystallised by slow evaporation from toluene.[97,102] In some case it was concomitant with form III and VII. The fact that it was never observed during experiments mentioned above might suggest that crystallisation of form IX is difficult ($\bar{R}^N_{depth}$[IX]=-0.204) and we note that it is also the form with the largest rugosity.

Table S3. Calculated $\bar{R}^N_{depth}(\text{Å})$ for polymorphs of acridine.

Acridine	REFCODE	Form	$\bar{R}^N_{depth}$	$\Delta\bar{R}^N_{depth}[eh]$
	ACRDIN12	IX	-0.204	0.128
	ACRDIN06	VII	-0.160	0.084
	ACRDIN05	VI	-0.158	0.082
	ACRDIN08	IV	-0.174	0.098
	ACRDIN07	III	-0.117	0.041
	ACRDIN04	II	-0.076	0.000

S4.3. Coumarin

Coumarin is a small organic molecule known to crystallise as five polymorphs (forms I-V). Although evidence of polymorphism of coumarin has been known since 1929, it is interesting to note that until very recently, only the crystal structure of form I was available. In 2017, Shtukenberg et al.[103] solved the crystal structure of four polymorphic forms (forms I, III-V). Among the five polymorphs of coumarin, only form I can be easily crystallised from solvents,[104,105] all the other polymorphs has been obtained directly from the melt or as result of phase transformations.[103] Forms II-V transforms all rapidly into form I. The order of stability of I-V, based on experimental results, is the following: I > II > III > IV > V. When we analyse the calculated rugosities, it is interesting to note that form I is the most stable and has the second smallest values ($\bar{R}^N_{depth}$[I]= -0.134). The smoothest value is observed for the monoclinic form II ($\bar{R}^N_{depth}[II]$= -0.044). The fact that form II was only recently obtained might be explained considering that, even if it nucleates faster, its observation could have been prevented by its quick transformation into I. In 1934 Kofler and Geyr observed two polymorphs of coumarine and, on the basis of optical measurements, identified one

of them as monoclinic.[106] All forms III, IV and V have larger rugosity values than forms I and II, consistent with the fact that they are also harder crystallise (table S3).

Table S4. Calculated $\bar{R}^{N}_{depth}$(Å) for polymorphs I-V of coumarine.

Coumarin Stability Order	REFCODE	Form	$\bar{R}^{N}_{depth}$	$\Delta\bar{R}^{N}_{depth}[eh]$
#5	COUMAR18	V	-0.204	0.160
#4	COUMAR16	IV	-0.209	0.165
#3	COUMAR15	III	-0.146	0.102
#2	COUMAR13	II	-0.044	0.000
#1	COUMAR11	I	-0.134	0.090

*1 being the most stable and 5 the least stable.

S4.4. ROY

5-Methyl-2-[(2-nitrophenyl)amino]-3-thiophenecarbonitrile, also known as ROY, represents an interesting case of conformational polymorphism and, as a consequence, the object of different experimental[107–109] and computational studies.[107–111] Due to its conformational flexibility and to the different degree of conjugation between the aromatic rings in the molecular skeleton polymorphs of ROY adopt different colours (red, orange and yellow). Eleven polymorphic forms (Y, ON, R, OP, YN, ORP, Y04, YT04, R05, RPL and P013) have been so far isolated, eight of which (Y, ON, R, OP, YN, ORP, YT04 and R05) have been fully structurally characterised by single crystal X-ray diffraction. [108,112,113]Recently, the crystal structure of PO13 was obtained from PXRD data. [109]

Most of ROY polymorphs can be obtained concomitantly from the same liquid and are kinetically stable at ambient-conditions.[107] Among the nine structurally characterised polymorphs, only R, Y, ON, OP, YN, YT04 and ORP have been directly obtained by crystallisation from solutions. R was obtained from ethanol concomitantly with Y.[113] ON and YN were produced by fast crystallisations, OP was obtained concomitantly with ON by seeding experiments. [112] YT04 was initially obtained as the product of a transformation from Y04; single crystals have been then isolated by seeding a saturated solution of ROY with YT04.[114] The most stable polymorph Y was obtained by solution mediated transformation of the other polymorphs at room temperature.[112] The recently discovered R05 was obtained from the melt by cross-nucleation on Y04. like R05, PO13 was obtained from the melt during a variable temperature PXRD experiment, starting from YN.[109] The calculated rugosities (see Table S5) suggests that YN, R and ON are smoother polymorphs when compared to the others which is consistent with the fact that they are also easier to obtain.

Table S5. Calculated $\bar{R}^{N}_{depth}$(Å) for polymorphs of ROY.

ROY	REFCODE	Form	$\bar{R}^{N}_{depth}$	$\Delta\bar{R}^{N}_{depth}[eh]$
	QAXMEH01	Y	-0.265	0.208
	QAXMEH03	OP	-0.250	0.193
	QAXMEH	ON	-0.101	0.044
	QAXMEH02	R	-0.102	0.045
	QAXMEH04	YN	-0.057	0.000
	QAXMEH12	YT04	-0.215	0.158
	QAXMEH05	ORP	-0.164	0.107
	CIF[109]	PO13	-0.119	0.062

S5. CSP Methods

The computer programme GRACE was used to generate an accurate lattice energy landscape of diflunisal and compound X. GRACE uses a dispersion-corrected density functional theory method (DFT-D) that combines calculations with the PBE functional in VASP. Every crystal structure prediction starts by fitting a tailor-made force field (TMFF) to DFT-D reference data. The actual crystal structure prediction is a convergence-controlled three-step procedure, executed separately for one and two molecules per asymmetric unit. In the first step, a large number of crystal structures are generated with a Monte Carlo parallel tempering algorithm using the tailor-made force field. In the second step, some of these structures are subjected to a coarse lattice energy optimisation at the DFT-D level. In the final step, a small number of structures are subjected to a more stringent DFT-D lattice energy optimisation. DFT calculations use a plane wave cut-off energy of 520 eV and a k-point spacing of roughly 0.07 $Å^{-1}$. All lattice energy minimisations of the final step are converged to within at least 0.003 Å for atomic displacements, 0.00025 kcal mol^{-1} per atom for energy changes, 0.7 kcal mol−1 Å−1 for atomic forces and 1.0 kbar for cell stress. In the second step the lattice energies are converged to within at least 0.02 Å for atomic displacements, 0.001 kcal mol−1 per atom for energy changes, 7.0 kcal mol−1 Å−1 for atomic forces and 15.0 kbar for cell stress. The convergence criteria of the final step were applied when performing the lattice energy minimisations in the disorder models.

S6. References

1. Bryant, M. J., Maloney, A. G. P. & Sykes, R. A. Predicting mechanical properties of crystalline materials through topological analysis. *CrystEngComm* **20**, 2698–2704 (2018).

2. Groom, C. R., Bruno, I. J., Lightfoot, M. P. & Ward, S. C. The Cambridge Structural Database. *Acta Crystallogr. Sect. B* **72**, 171–179 (2016).

3. Chisholm, J. A. & Motherwell, S. {\it COMPACK}: a program for identifying crystal structure similarity using distances. *J. Appl. Crystallogr.* **38**, 228–231 (2005).

4. Braun, D. E., Vickers, M. & Griesser, U. J. Dapsone Form V: A Late Appearing Thermodynamic Polymorph of a Pharmaceutical. *Mol. Pharm.* (2019) doi:10.1021/acs.molpharmaceut.9b00419.

5. Gavezzotti, A. & Filippini, G. Polymorphic Forms of Organic Crystals at Room Conditions: Thermodynamic and Structural Implications. *J. Am. Chem. Soc.* **117**, 12299–12305 (1995).

6. Payne, R. S., Rowe, R. C., Roberts, R. J., Charlton, M. H. & Docherty, R. Potential polymorphs of aspirin. *J. Comput. Chem.* **20**, 262–273 (1999).

7. Ouvrard, C. & Price, S. L. Toward crystal structure prediction for conformationally flexible molecules: The headaches illustrated by aspirin. *Cryst. Growth Des.* **4**, 1119–1127 (2004).

8. Vishweshwar, P., McMahon, J. A., Oliveira, M., Peterson, M. L. & Zaworotko, M. J. The predictably elusive form II of aspirin. *J. Am. Chem. Soc.* **127**, 16802–16803 (2005).

9. Bond, A. D., Boese, R. & Desiraju, G. R. On the polymorphism of aspirin: Crystalline aspirin as intergrowths of two 'polymorphic' domains. *Angew. Chemie - Int. Ed.* **46**, 618–622 (2007).

10. Bond, A. D., Solanko, K. A., Parsons, S., Redder, S. & Boese, R. Single crystals of aspirin form II: Crystallisation and stability. *CrystEngComm* **13**, 399–401 (2011).

11. Crowell, E. L., Dreger, Z. A. & Gupta, Y. M. High-pressure polymorphism of acetylsalicylic acid (aspirin): Raman spectroscopy. *J. Mol. Struct.* **1082**, 29–37 (2015).

12. Shtukenberg, A. G. *et al.* The Third Ambient Aspirin Polymorph. *Cryst. Growth Des.* **17**, 3562–3566 (2017).

13. Kuz'mina, L. G., Struchkov, Y. T., Novozhilova, N. V., and Tudorovskaya, G. L. Crystal structure of an aqueous solvate of p-diaminodiphenylsulfone. *Kristallografiya* **26**, 695–701 (1981).

14. Bel'Skii, V.K., Chernikova, N.Y., Rotaru, V.K., Kruchinin, M. M. Crystal and molecular structure of components of epoxy resins. II. 4,4'-Diaminodiphenyl sulfone hydrateNo Title. *Kristallografiya* **28**, 690 (1983).

15. Lemmer, H., Stieger, N., Liebenberg, W. & Caira, M. R. Solvatomorphism of the antibacterial dapsone: X-ray structures and thermal desolvation kinetics. *Cryst. Growth Des.* **12**, 1683–1692 (2012).

16. Braun, D. E. Experimental and computational approaches to rationalise multicomponent supramolecular assemblies: Dapsone monosolvates. *Phys. Chem. Chem. Phys.* **21**, 17288–17305 (2019).

17. Braun, D. E., Krüger, H., Kahlenberg, V. & Griesser, U. J. Molecular Level Understanding of the Reversible Phase Transformation between Forms III and II of Dapsone. *Cryst. Growth Des.* **17**, 5054–5060 (2017).

18. Chekal, B. P. *et al.* The Challenges of Developing an API Crystallization Process for a Complex Polymorphic and Highly Solvating System. Part I. *Org. Process Res. Dev.* **13**, 1327–1337 (2009).

19. Campeta, A. M. *et al.* Development of a targeted polymorph screening approach for a complex polymorphic and highly solvating API. *J. Pharm. Sci.* **99**, 3874–3886 (2010).

20. Abramov, Y. A. QTAIM application in drug development: Prediction of relative stability of drug polymorphs from experimental crystal structures. *J. Phys. Chem. A* **115**, 12809–12817 (2011).

21. Vasileiadis, M., Pantelides, C. C. & Adjiman, C. S. Prediction of the crystal structures of axitinib, a polymorphic pharmaceutical molecule. *Chem. Eng. Sci.* **121**, 60–76 (2015).

22. Sullivan, R. A. & Davey, R. J. Concerning the crystal morphologies of the α and β polymorphs of p-aminobenzoic acid. *CrystEngComm* **17**, 1015–1023 (2015).

23. Rosbottom, I., Roberts, K. J. & Docherty, R. The solid state, surface and morphological properties of p-aminobenzoic acid in terms of the strength and directionality of its intermolecular synthons. *CrystEngComm* **17**, 5768–5788 (2015).

24. Cruz-Cabeza, A. J., Davey, R. J., Oswald, I. D. H., Ward, M. R. & Sugden, I. J. Polymorphism in p-

aminobenzoic acid. *CrystEngComm* **21**, 2034–2042 (2019).

25. Svärd, M., Nordström, F. L., Hoffmann, E.-M., Aziz, B. & Rasmuson, Å. C. Thermodynamics and nucleation of the enantiotropic compound p-aminobenzoic acid. *CrystEngComm* **15**, 5020–5031 (2013).

26. Li, Z. & Matzger, A. J. Influence of Coformer Stoichiometric Ratio on Pharmaceutical Cocrystal Dissolution: Three Cocrystals of Carbamazepine/4-Aminobenzoic Acid. *Mol. Pharm.* **13**, 990–995 (2016).

27. Cherukuvada, S., Babu, N. J. & Nangia, A. Nitrofurantoin-p-aminobenzoic acid cocrystal: Hydration stability and dissolution rate studies. *J. Pharm. Sci.* **100**, 3233–3244 (2011).

28. Benali-Cherif, R., Takouachet, R., Bendeif, E.-E. & Benali-Cherif, N. The structural properties of a noncentrosymmetric polymorph of 4-amino{\-}benzoic acid. *Acta Crystallogr. Sect. C* **70**, 323–325 (2014).

29. Ward, M. R. *et al.* Discovery and recovery of delta p-aminobenzoic acid. *CrystEngComm* **21**, 2058–2066 (2019).

30. Gracin, S., Uusi-Penttilä, M. & Rasmuson, Å. C. Controlling polymorphism of p-aminobenzoic acid by sonication. *VDI Berichte* 677–682 (2005).

31. Zhang, T., Szilágyi, B., Gong, J. & Nagy, Z. K. Thermodynamic Polymorph Selection in Enantiotropic Systems Using Supersaturation-Controlled Batch and Semibatch Cooling Crystallization. *Cryst. Growth Des.* **19**, 6715–6726 (2019).

32. Hursthouse, M. B., Montis, R. & Tizzard, G. J. Intriguing relationships and associations in the crystal structures of a family of substituted aspirin molecules. *CrystEngComm* **12**, 953–959 (2010).

33. Solanko, K. A. & Bond., A. D. Influence of impurities on the crystallisation of 5-X-aspirin and 5-X-aspirin anhydride polymorphs (X = Cl, Br, Me). *CrystEngComm* **13**, 6991–6996 (2011).

34. Childs, S. L., Wood, P. A., Rodríguez-Hornedo, N., Reddy, L. S. & Hardcastle, K. I. Analysis of 50 crystal structures containing carbamazepine using the Materials module of Mercury CSD. *Cryst. Growth Des.* **9**, 1869–1888 (2009).

35. Arlin, J.-B., Price, L. S., Price, S. L. & Florence, A. J. A strategy for producing predicted polymorphs: Catemeric carbamazepine form v. *Chem. Commun.* **47**, 7074–7076 (2011).

36. Childs, S. L. *et al.* Screening strategies based on solubility and solution composition generate pharmaceutically acceptable cocrystals of carbamazepine. *CrystEngComm* **10**, 856–864 (2008).

37. Fleischman, S. G. *et al.* Crystal Engineering of the Composition of Pharmaceutical Phases: Multiple-Component Crystalline Solids Involving Carbamazepine. *Cryst. Growth Des.* **3**, 909–919 (2003).

38. Grzesiak, A. L., Lang, M., Kim, K. & Matzger, A. J. Comparison of the Four Anhydrous Polymorphs of Carbamazepine and the Crystal Structure of Form I. *J. Pharm. Sci.* **92**, 2260–2271 (2003).

39. Lowes, M. M. J., Caira, M. R., Lotter, A. P. & Van Watt, J. G. D. Physicochemical properties and x-ray structural studies of the trigonal polymorph of carbamazepine. *J. Pharm. Sci.* **76**, 744–752 (1987).

40. Himes, V. L., Mighell, A. D. & De Camp, W. H. Structure of carbamazepine: 5{\it H}-dibenz[{\it b},{\it f}]azepine-5-carboxamide. *Acta Crystallogr. Sect. B* **37**, 2242–2245 (1981).

41. Lang, M., Kampf, J. W. & Matzger, A. J. Form IV of Carbamazepine. *J. Pharm. Sci.* **91**, 1186–1190 (2002).

42. Krahn, F. U. & Mielck, J. B. Effect of type and extent of crystalline order on chemical and physical stability of carbamazepine. *Int. J. Pharm.* **53**, 25–34 (1989).

43. Harris, R. K. *et al.* Structural Studies of the Polymorphs of Carbamazepine, Its Dihydrate, and Two Solvates. *Org. Process Res. Dev.* **10**, 165 (2006).

44. Diao, Y. *et al.* Gel-Induced Selective Crystallization of Polymorphs. *J. Am. Chem. Soc.* **134**, 673–684 (2012).

45. Kelly, R. C. & Rodríguez-Hornedo, N. Solvent effects on the crystallization and preferential nucleation of carbamazepine anhydrous polymorphs: A molecular recognition perspective. *Org. Process Res. Dev.* **13**, 1291–1300 (2009).

46. Vrcelj, R. M. *et al.* Two new paracetamol/dioxane solvates - A system exhibiting a reversible solid-state phase transformation. *J. Pharm. Sci.* **92**, 2069–2073 (2003).

47. Haisa M, Kashino S, Kawaii R, M. H. The monoclinic form of p-hydroxyacetanilide. *Acta Crystallogr* **B32**, 1283–1285 (1976).

48. Haisa M, Kashino S, M. H. The orthorhombic form of p-hydroxyacetanilide. *Acta Crystallogr* **B30**, 2510–2512 (1974).

49. Perrin, M.-A.; Neumann, M. A.; Elmaleh, H.; Zaske, L. Crystal structure determination of the elusive paracetamol Form III. *Chem.Commun.* **22**, 3181−3183 (2009).

50. Reiss, C. A.; van Mechelen, J. B.; Goubitzc, K.; Peschar, R. Reassessment of paracetamol orthorhombic Form III and determination of a novel lowtemperaturemonoclinic Form III-m from powder diffraction data. ,. *Acta Crystallogr.Sect. C Struct. Chem.* **74**, 392−399 (2018).

51. Shtukenberg, A. G. *et al.* Melt Crystallization for Paracetamol Polymorphism. *Cryst. Growth Des.* **19**, 4070–4080 (2019).

52. Smith, S. J., Bishop, M. M., Montgomery, J. M., Hamilton, T. P. & Vohra, Y. K. Polymorphism in paracetamol: Evidence of additional forms IV and v at high pressure. *J. Phys. Chem. A* **118**, 6068–6077 (2014).

53. Nicnols, G. & Frampton, C. S. Physicochemical characterization of the orthorhombic polymorph of paracetamol crystallized from solution. *J. Pharm. Sci.* **87**, 684–693 (1998).

54. Peterson, M. L. *et al.* Crystallization and transformation of acetaminophen trihydrate. *Cryst. Growth Des.* **3**, 761–765 (2003).

55. Cruz, P. C., Rocha, F. A. & Ferreira, A. M. Application of Selective Crystallization Methods To Isolate the Metastable Polymorphs of Paracetamol: A Review. *Org. Process Res. Dev.* (2019) doi:10.1021/acs.oprd.9b00322.

56. McAfee, D. A., Hadgraft, J. & Lane, M. E. Rotigotine: The first new chemical entity for transdermal drug delivery. *Eur. J. Pharm. Biopharm.* **88**, 586–593 (2014).

57. Rietveld, I. B. & Céolin, R. Rotigotine: Unexpected Polymorphism with Predictable Overall Monotropic Behavior. *J. Pharm. Sci.* **104**, 4117–4122 (2015).

58. Mortazavi, M. *et al.* Author Correction: Computational polymorph screening reveals late-appearing and poorly-soluble form of rotigotine (Communications Chemistry, (2019), 2, 1, (70), 10.1038/s42004-019-0171-y). *Commun. Chem.* **2**, (2019).

59. Suresh, K. & Nangia, A. Curcumin: Pharmaceutical solids as a platform to improve solubility and bioavailability. *CrystEngComm* **20**, 3277–3296 (2018).

60. Chakrabarti, R., Rawat, P. S., Cooke, B. M., Coppel, R. L. & Patankar, S. Cellular Effects of Curcumin on Plasmodium falciparum Include Disruption of Microtubules. *PLoS One* **8**, 1–14 (2013).

61. Tønnesen, Hanne Hjorth; Karlsen, Jan; Mostad, A. Structural Studies of Curcuminoids. I. The Crystal Structure of Curcumin. *Acta Chem. Scand.* **36b**, 475–479 (1982).

62. Sanphui, P., Goud, N. R., Khandavilli, U. B. R., Bhanoth, S. & Nangia, A. New polymorphs of curcumin. *Chem. Commun.* **47**, 5013–5015 (2011).

63. Sanphui, P., Goud, N. R., Khandavilli, U. B. R. & Nangia, A. Fast dissolving curcumin cocrystals. *Cryst. Growth Des.* **11**, 4135–4145 (2011).

64. T.De Silva, I.M.Warner, F. R. F. *private communication*.

65. Thorat, A. A. & Dalvi, S. V. Solid-state phase transformations and storage stability of curcumin polymorphs. *Cryst. Growth Des.* **15**, 1757–1770 (2015).

66. Thorat, A. A. & Dalvi, S. V. Particle formation pathways and polymorphism of curcumin induced by ultrasound and additives during liquid antisolvent precipitation. *CrystEngComm* **16**, 11102–11114 (2014).

67. Kurniawansyah, F., Mammucari, R. & Foster, N. R. Polymorphism of curcumin from dense gas antisolvent precipitation. *Powder Technol.* **305**, 748–756 (2017).

68. Bobrovs, R., Seton, L. & Dempster, N. The reluctant polymorph: Investigation into the effect of self-association on the solvent mediated phase transformation and nucleation of theophylline. *CrystEngComm* **17**, 5237–5251 (2015).

69. Sun, C., Zhou, D., Grant, D. J. W. & Young Jr, V. G. Theophyl{\-}line monohydrate. *Acta Crystallogr. Sect. E* **58**, o368--o370 (2002).

70. Cardin, C., Gan, Y. & Lewis, T. Low-temperature determination of theophylline dimethyl sulfoxide solvate. *Acta Crystallogr. Sect. E* **63**, o3175 (2007).

71. Fucke, K. *et al.* New insights into an old molecule: Interaction energies of theophylline crystal forms. *Cryst. Growth Des.* **12**, 1395–1401 (2012).

72. Koranne, S., Krzyzaniak, J. F., Luthra, S., Arora, K. K. & Suryanarayanan, R. Role of Coformer and Excipient Properties on the Solid-State Stability of Theophylline Cocrystals. *Cryst. Growth Des.* **19**, 868–875 (2019).

73. Trask, A. V, Motherwell, W. D. S. & Jones, W. Physical stability enhancement of theophylline via cocrystallization. *Int. J. Pharm.* **320**, 114–123 (2006).

74. Khamar, D., Bradshaw, I. J., Hutcheon, G. A. & Seton, L. Solid state transformations mediated by a kinetically stable form. *Cryst. Growth Des.* **12**, 109–118 (2012).

75. Seton, L., Khamar, D., Bradshaw, I. J. & Hutcheon, G. A. Solid state forms of theophylline: Presenting a new anhydrous polymorph. *Cryst. Growth Des.* **10**, 3879–3886 (2010).

76. Khamar, D., Pritchard, R. G., Bradshaw, I. J., Hutcheon, G. A. & Seton, L. Polymorphs of anhydrous theophylline: Stable form IV consists of dimer pairs and metastable form i consists of hydrogen-bonded chains. *Acta Crystallogr. Sect. C Cryst. Struct. Commun.* **67**, o496–o499 (2011).

77. Ebisuzaki, Y., Boyle, P. D. & Smith, J. A. Methylxanthines. I. Anhydrous Theophylline. *Acta Crystallogr. Sect. C* **53**, 777–779 (1997).

78. Suzukal, E., Kikuko shimomura, K. & Sekiguchi, K. Thermochemical Study of Theophylline and Its Hydrate. *Chem. Pharm. Bull.* **37**, 493–497 (1989).

79. Bučar, D.-K., Lancaster, R. W. & Bernstein, J. Disappearing Polymorphs Revisited. *Angew. Chemie Int. Ed.*

54, 6972–6993 (2015).

80. Dunitz, J. D. & Bernstein, J. Disappearing Polymorphs. *Acc. Chem. Res.* **28**, 193–200 (1995).

81. Chemburkar, S. R. *et al.* Dealing with the impact of ritonavir polymorphs on the late stages of bulk drug process development. *Org. Process Res. Dev.* **4**, 413–417 (2000).

82. Bauer, J. *et al.* Ritonavir: An extraordinary example of conformational polymorphism. *Pharm. Res.* **18**, 859–866 (2001).

83. Chakraborty, D., Sengupta, N. & Wales, D. J. Conformational Energy Landscape of the Ritonavir Molecule. *J. Phys. Chem. B* **120**, 4331–4340 (2016).

84. Morissette, S. L., Soukasene, S., Levinson, D., Cima, M. J. & Almarsson, Ö. Elucidation of crystal form diversity of the HIV protease inhibitor ritonavir by high-throughput crystallization. *Proc. Natl. Acad. Sci. U. S. A.* **100**, 2180–2184 (2003).

85. Bernal, J. D. The Crystal Structure of the Natural Amino Acids and Related Compounds. *Z. Krist.* **78**, 363–369 (1931).

86. Iitaka, Y. The crystal structure of β-glycine. *Acta Crystallogr.* **13**, 35–45 (1960).

87. Iitaka, Y. The crystal structure of γ-glycine. *Acta Crystallogr.* **11**, (1958).

88. Bull, C. L. *et al.* {$ζ$}-Glycine: insight into the mechanism of a polymorphic phase transition. *IUCrJ* **4**, 569–574 (2017).

89. Hughes, C. E., Hamad, S., Harris, K. D. M., Catlow, C. R. A. & Griffiths, P. C. A multi-technique approach for probing the evolution of structural properties during crystallization of organic materials from solution. *Faraday Discuss.* **136**, 71–89 (2007).

90. Weissbuch, I., Torbeev, V. Y., Leiserowitz, L. & Lahav, M. Solvent Effect on Crystal Polymorphism: Why Addition of Methanol or Ethanol to Aqueous Solutions Induces the Precipitation of the Least Stable β Form of Glycine. *Angew. Chemie Int. Ed.* **44**, 3226–3229 (2005).

91. Bouchard, A., Hofland, G. W. & Witkamp, G.-J. Solubility of Glycine Polymorphs and Recrystallization of β-Glycine. *J. Chem. Eng. Data* **52**, 1626–1629 (2007).

92. Weissbuch, I., Leisorowitz, L. & Lahav, M. "Tailor-Made" and charge-transfer auxiliaries for the control of the crystal polymorphism of glycine. *Adv. Mater.* **6**, 952–956 (1994).

93. Iitaka, Y. The crystal structure of γ-glycine. *Acta Crystallogr.* **14**, 1–10 (1961).

94. Towler, C. S., Davey, R. J., Lancaster, R. W. & Price, C. J. Impact of Molecular Speciation on Crystal Nucleation in Polymorphic Systems: The Conundrum of γ Glycine and Molecular 'Self Poisoning''. *J. Am. Chem. Soc.* **126**, 13347–13353 (2004).

95. Vesga, M. J., McKechnie, D., Mulheran, P. A., Johnston, K. & Sefcik, J. Conundrum of γ glycine nucleation revisited: to stir or not to stir? *CrystEngComm* **21**, 2234–2243 (2019).

96. Chew, J. W., Black, S. N., Chow, P. S., Tan, R. B. H. & Carpenter, K. J. Stable polymorphs: difficult to make and difficult to predict. *CrystEngComm* **9**, 128–130 (2007).

97. Schur, E. *et al.* The (Current) Acridine Solid Form Landscape: Eight Polymorphs and a Hydrate. *Cryst. Growth Des.* **19**, 4884–4893 (2019).

98. Clarke, B. P., Thomas, J. M. & Williams, J. O. The relationship between crystallographic structure and luminescence in single crystals of acridine. *Chem. Phys. Lett.* **35**, 251–254 (1975).

99. Mei, X. & Wolf, C. Formation of new polymorphs of acridine using dicarboxylic acids as crystallization templates in solution. *Cryst. Growth Des.* **4**, 1099–1103 (2004).

100. Braga, D.; Grepioni, F.; Maini, L.; Mazzeo, P. P.; Rubini, K. Solvent-free preparation of co-crystals of phenazine and acridine with vanillin. *Thermochim. Acta* 507−508 (2010).

101. Musumeci, D., Hunter, C. A. & McCabe, J. F. Solvent effects on acridine polymorphism. *Cryst. Growth Des.* **10**, 1661–1664 (2010).

102. Stephens, P. W., Schur, E., Lapidus, S. H. & Bernstein, J. Acridine form IX. *Acta Crystallogr. Sect. E Crystallogr. Commun.* **75**, 489–491 (2019).

103. Shtukenberg, A. G. *et al.* Powder diffraction and crystal structure prediction identify four new coumarin polymorphs. *Chem. Sci.* **8**, 4926–4940 (2017).

104. Munshi, P. & Guru Row, T. N. Exploring the Lower Limit in Hydrogen Bonds: Analysis of Weak C−H···O and C−H···π Interactions in Substituted Coumarins from Charge Density Analysis. *J. Phys. Chem. A* **109**, 659–672 (2005).

105. Hsieh, T.-J., Su, C.-C., Chen, C.-Y., Liou, C.-H. & Lu, L.-H. Using experimental studies and theoretical calculations to analyze the molecular mechanism of coumarin, p-hydroxybenzoic acid, and cinnamic acid. *J. Mol. Struct.* **741**, 193–199 (2005).

106. Kofler, A. & Geyr, J. Zum mikrochemischen Nachweis des Cumarins. *Mikrochemie* **15**, 67–73 (1934).

107. Yu, L. Polymorphism in molecular solids: An extraordinary system of red, orange, and yellow crystals. *Acc. Chem. Res.* **43**, 1257–1266 (2010).

108. Tan, M. *et al.* ROY revisited, again: the eighth solved structure. *Faraday Discuss.* **211**, 477–491 (2018).

109. Gushurst, K. S., Nyman, J. & Boerrigter, S. X. M. The PO13 crystal structure of ROY. *CrystEngComm* **21**, 1363–1368 (2019).

110. Vasileiadis, M., Kazantsev, A. V, Karamertzanis, P. G., Adjiman, C. S. & Pantelides, C. C. The polymorphs of ROY: Application of a systematic crystal structure prediction technique. *Acta Crystallogr. Sect. B Struct. Sci.* **68**, 677–685 (2012).

111. Nyman, J., Yu, L. & Reutzel-Edens, S. M. Accuracy and reproducibility in crystal structure prediction: the curious case of ROY. *CrystEngComm* **21**, 2080–2088 (2019).

112. Yu, L. *et al.* Thermochemistry and conformational polymorphism of a hexamorphic crystal system. *J. Am. Chem. Soc.* **122**, 585–591 (2000).

113. Stephenson, G. A. *et al.* Conformational and color polymorphism of 5-methyl-2-[(2-nitrophenyl)amino]-3-thiophenecarbonitrile. *J. Pharm. Sci.* **84**, 1385–1386 (1995).

114. Chen, S., Guzei, I. A. & Yu, L. New polymorphs of ROY and new record for coexisting polymorphs of solved structures. *J. Am. Chem. Soc.* **127**, 9881–9885 (2005).
